# The Relationship between Childhood Trauma and Depression in Early Adulthood: The Roles of Resilience and Personality Type

**DOI:** 10.62641/aep.v53i2.1690

**Published:** 2025-03-05

**Authors:** Binbin Wang, Jingyi Zhang, Chengqi Cao, Ling Xu, Mingyue Gao, Qin Zhang, Kunlin Zhang

**Affiliations:** ^1^CAS Key Laboratory of Mental Health, Institute of Psychology, Chinese Academy of Sciences, 100101 Beijing, China; ^2^Department of Psychology, University of Chinese Academy of Sciences, 100049 Beijing, China; ^3^Shanghai Mental Health Center, Shanghai Jiao Tong University School of Medicine, 200030 Shanghai, China

**Keywords:** childhood trauma, depression, RUO personality types, resilience, latent profile analysis

## Abstract

**Background:**

The relationship between childhood trauma and depression in early adulthood is complex and influenced by factors such as resilience and personality type. This study aims to investigate the mediating role of resilience and the moderating role of personality types in this relationship.

**Methods:**

A total of 1059 undergraduates (mean age = 19.87 ± 1.82 years; 48.1% men, 51.9% women) were surveyed. The Big Five Personality Inventory (BFI) was used to assess the personality dimensions of the participants, which were further analyzed using latent profile analysis (LPA). Childhood trauma experiences were evaluated using the short form of the Childhood Trauma Questionnaire (CTQ), while resilience was measured using the Connor-Davidson Resilience Scale (CD-RISC). Depressive symptoms were assessed using the Center for Epidemiological Studies-Depression Scale (CES-D). All scales demonstrated high reliability and validity.

**Results:**

The findings indicated a positive correlation between childhood trauma and depression, mediated by resilience. Personality types moderated this mediation, with significant indirect effects observed only for individuals categorized as Type 2.

**Conclusions:**

This study provides insight into the mechanisms of depression in early adulthood, suggesting that an intervention targeting resilience and considering personality type may be beneficial. The result highlight the importance of a human-centered approach in understanding the interaction among personality traits and their potential moderating effect on the relationship between childhood trauma and depression symptoms.

## Introduction

Childhood upbringing, as an early life environmental factor, is strongly associated with mental health status. Depression emerges as a prevalent mental health concern and is closely linked to experiences of trauma during developmental years [1]. Depression, characterized by diminished interest, impaired cognitive functioning, and mood disturbances, has a lifetime prevalence rate of 13.2% [2]. The report estimates that 5% of adults worldwide suffer from depression each year [3]. According to the latest national epidemiological survey of mental disorders, the lifetime prevallence rate of depressive disorder in Chinese adults is 6.8%, and the 12-month prevalence rate is 3.6%, which is lower than the world average, and the prevalence rate in women is higher than that in men [4].

A meta-analysis suggests that over 50% of depression cases globally may be attributed to self-reported traumatic childhood experiences [5]. A study has reported that higher levels of childhood trauma are associated with more severe depressive symptoms [6]. Researchers have identified emotional mistreatment and neglect as significant contributors to depressive disorders [7]. Childhood trauma leads to significant clinical and neurobiological differences in major depressive disorder [8].

While the link between childhood trauma and depressive symptoms in early adulthood has been extensively studied, there is limited research on the psychological mechanisms through which individuals cope with these traumas and develop depression. This study aimed to provide a new perspective by exploring the mediating role of resilience and the moderating role of personality types. The findings of this study have significant practical implications for early identification, prevention, and intervention of depression in early adulthood, offering a new theoretical framework and direction for future research. Consequently, the following hypothesis was proposed:

H1: Childhood trauma is positively associated with depression.

### The Mediating Role of Resilience

Although childhood trauma is directly related to depression, not every individual who experiences childhood trauma develops depression. Individuals with different abilities (e.g., resilience) will recover from childhood trauma to different degrees. Adults who experienced childhood trauma had significantly higher rates of depression in adulthood compared to those who did not experience trauma, and mental resilience had a moderate inverse relationship with depression [9, 10]. Meta-analyses have shown that psychological resilience significantly reduced the association between childhood trauma and depressive symptoms, with no significant differences in depressive symptoms between high- and low-resilience individuals in regard to trauma history [11].

Resilience is a protective factor for the mental health of an individual [12]. Cross-sectional study have demonstrated that resilience is negatively associated with depressive symptoms [13], and longitudinal study have shown that the level of resilience of an individual negatively predicts the onset of depression [14]. Furthermore, resilience is dynamic and changes with the environment; negative environment, such as childhood trauma, can reduce the resilience of an individual [15]. Decreased resilience is associated with elevated depressive symptoms [13]. The pathway model suggests that 39.8% of the association between childhood abuse and suicidal ideation is mediated by psychological resilience [16]. Numerous studies have shown that childhood trauma directly affects depressive symptoms and indirectly affects them through the mediating pathway of psychological resilience. Improving psychological resilience to cope with daily stress is an effective clinical intervention strategy to reduce the impact of childhood trauma on depression [17, 18, 19]. Therefore, we further proposed the following hypothesis:

H2: Resilience acts as a mediator between the experience of childhood trauma and the onset of depression.

### The Moderating Role of Personality Type

Personality traits are stable psychological characteristics that influence the thoughts, feelings, and behaviours of an individual. The Big Five personality theory identifies five core personality traits: openness, extroversion, conscientiousness, agreeableness, and neuroticism [20]. Models of personality processes suggest that specific personality traits can moderate the link between childhood adversity and the development of depressive symptoms. Investigations into personality have historically employed either a variable-centric framework or an individual-centric perspective [21, 22]. Experts propose that the person-centered approach focuses on how individual characteristics are structured and unified, blending the interplay among various personality facets [23]. As childhood trauma affects the whole individual rather than isolated traits, we adopted a person-centered methodology for this research.

Based on theories of self-resilience and self-control, previous studies have recognized three typical personality types, referred to as the three common personality types: resilient, over-controlled, and under-controlled (RUO types) [24, 25, 26, 27]. Simple multivariate normal distributions with a large five-correlation structure can generate RUO types using three clustering methods [28]. The resilient personality is characterized by low neuroticism, high conscientiousness, moderately high agreeableness, high openness, and significant extroversion. Those with an over-controlled personality typically exhibit high agreeableness, low extroversion, and elevated neuroticism. In contrast, individuals with an under-controlled personality typically display low agreeableness and a lack of conscientiousness [24].

Previous research indicates that the onset of depression during early adulthood tends to correlate positively with experiences of childhood adversity and the personality trait of neuroticism, while it exhibits a negative correlation with the trait of extroversion [29, 30]. Individuals scoring high in neuroticism exhibit reduced regulation of the amygdala by the anterior cingulate cortex, a condition linked to affective disorders, including depression and anxiety [31]. Childhood trauma, neuroticism, and low resilience are significantly associated with depressive symptoms in various populations [32]. The study found that childhood trauma positively predicts depressive symptoms in early adulthood, with neuroticism and psychological resilience serving as partial mediators in this relationship [30]. Individuals with a resilient personality have the lowest levels of depression compared to the other two personality types. Research on personality traits has demonstrated that individuals with resilience tend to be well-adapted in various aspects of life, exhibiting high pro-social behaviours and fewer mental health problems, and have the lowest levels of depression compared to others facing similar social situations. By comparison, individuals with an over-controlled personality type have a higher negative affect and a higher prevalence of mood disorders, whereas those with an under-controlled personality type exhibit higher levels of all maladaptive traits except disinhibition [27]. Consequently, we proposed the following hypothesis:

H3: The personality types of Chinese college students may be divided into different types, and distinct personality types may regulate the relationship between childhood trauma, resilience, and depressive symptoms.

### Current Research

Childhood trauma is a risk factor for the onset of depression in early adulthood, while resilience acts as a protective factor for mental well-being and a potential mechanism linking trauma to depression. Personality has been shown to have a moderating effect on this relationship, but there have been no studies directly examining the moderating effect of personality type on the relationships among childhood trauma, resilience, and depression despite some evidence supporting this potential role. The study aimed to examine the moderating role of different personality types and resilience in the link between childhood trauma and early depression.

## Materials and Methods

### Participants

A total of 1064 Chinese college students were recruited for this study through online and offline methods from 1 November 2021 to 1 January 2022. Among them, 544 online questionnaires were collected nationwide through the Questionnaire Star platform, and 520 paper questionnaires were collected offline through centralized administration at two colleges in Jilin Province, China. The inclusion criteria were: (1) college students aged 18–25; (2) voluntary participation in the survey. The exclusion criteria involved attention screening questions randomly set in the questionnaire. For example, participants were instructed to select the “completely inconsistent” option for specific questions. Those who did not choose the correct option were defined as inattentive. Inattentive subjects who submitted paper questionnaires were eliminated using five attention screening questions, while the online questionnaire had six such questions and was eliminated if any of them were answered incorrectly. We gathered 1059 actionable surveys, representing a 94.22% effectiveness rate. The age of the participants averaged 19.87 years (standard deviations (SD) = 1.82), with a gender distribution of 509 males (48.1%) and 550 females (51.9%).

### Measurements

#### Big Five Personality Inventory (BFI)

The Chinese version of the BFI [33] measured the five personality dimensions. This self-report scale consists of 44 items assessing the dimensions of openness (10 items; Cronbach’s = 0.794), conscientiousness (9 items; Cronbach’s = 0.775), extraversion (8 items; Cronbach’s = 0.752), agreeableness (9 items; Cronbach’s = 0.683), and neuroticism (8 items; Cronbach’s = 0.756). Participants were evaluated on a 5-point Likert scale, where 1 represents ‘*strongly disagree*’ and 5 stands for ‘*strongly agree*’. Additionally, 16 items were reverse-scored. Higher scores indicated more pronounced personality traits.

#### Childhood Trauma Questionnaire-Short Form (CTQ-SF)

The CTQ-SF, an abridged version of the Childhood Trauma Questionnaire, comprises 28 items that individuals use to retrospectively self-assess across five dimensions of potential childhood mistreatment: emotional maltreatment, physical maltreatment, sexual maltreatment, emotional neglect, and physical neglect [34]. Participants were queried about adverse experiences from their early years that occurred before age 16. Responses were rated on a scale from 1 (‘*never experienced*’) to 5 (‘*always experienced*’), with cumulative scores ranging from 25 to 125, where high scores reflect greater trauma exposure. The subscale cutoffs indicating the presence of trauma were as follows: Emotional Abuse at 13, Physical Abuse at 10, Sexual Abuse at 8, Emotional Neglect at 15, and Physical Neglect at 10. In this study, Emotional Abuse was detected in 33 respondents (3.1%), Emotional Neglect in 113 (10.7%), Physical Abuse in 36 (3.4%), Physical Neglect in 319 (30.1%), and Sexual Abuse in 65 (6.1%). A positive indication in any of the trauma subtypes defined overall childhood trauma, resulting in 396 participants (37.4%) being identified as having experienced childhood trauma. The overall scale demonstrated a Cronbach’s of 0.693. The specific subscales had the following Cronbach’s values: Emotional Abuse (0.687), Emotional Neglect (0.776), Physical Abuse (0.799), Physical Neglect (0.425), and Sexual Abuse (0.819).

#### Connor-Davidson Resilience Scale (CD-RISC)

The Connor-Davidson Resilience Scale (CD-RISC) was employed to measure the resilience of the participants. This scale [35], consists of 25 items divided across three subscales: optimism, resilience, and strength. Participants responded on a Likert-type scale ranging from 1 (‘not at all’) to 4 (‘fully’), with higher scores indicating greater resilience. In this study, the overall Cronbach’s α for the scale was 0.915, indicating excellent internal consistency. For the individual subscales, Cronbach’s α values were 0.860 for the resilience subscale, 0.808 for the self-reliance subscale, and 0.579 for the optimism subscale.

#### Center for Epidemiological Studies-Depression Scale (CES-D) 

The CES-D scale, developed by the National Institute of Mental Health, is primarily used to evaluate depressive symptoms during the current week, focusing on depressed affect or depressive states of mind [36]. This widely recognized tool assesses the severity of depressive symptoms without providing a clinical diagnosis. It has demonstrated good reliability and validity among Chinese college students [37, 38]. The CES-D is a 20-item self-reporting instrument that classifies individuals based on their total scores: scores of 9 or below suggest the absence of depressive symptoms, scores of 10 to 16 indicate mild depressive symptoms, scores of 17 to 24 suggest moderate depressive symptoms, and scores above 24 reflect moderate to severe depressive symptoms. The scale evaluates four dimensions: depressed mood, positive mood, physical symptoms, slowed activity, and interpersonal difficulties. Participants rated the frequency of these feelings over the past week using a four-point scale (0–3), where 0 indicates ‘less than one day’, 1 indicates ‘one to two days’, 2 indicates ‘three to four days’, and 3 indicates ‘five to seven days’. The instrument demonstrates solid reliability and validity in various cultural settings [39]. In this study, the Cronbach’s alpha coefficient of the scale was 0.788. Among the participants, 63 (6.0%) reported no depressive symptoms, 422 (39.8%) reported mild depressive symptoms, 351 (33.1%) reported moderate depressive symptoms, and 192 (18.1%) reported severe depressive symptoms.

### Data Processing

Data processing involved using EXCEL (Microsoft Excel 2021, Microsoft Corporation, Redmond, WA, USA) and the ‘careless’ package in RStudio to remove questionnaires that were not carefully answered. SPSS 22.0 (International Business Machines Corporation, Armonk, NY, USA) was used for reliability analyses, common method bias tests, descriptive statistics, and correlation analyses. The bias-corrected nonparametric percentile Bootstrap method in the SPSS plug-in PROCESS 3.5 was used to test moderated mediation effects. Latent profile analysis (LPA) was performed using Mplus 8.3 (Muthén & Muthén, Los Angeles, CA, USA).

### Data Analysis

Data analysis was performed using SPSS 22.0 and Mplus 8.3 software. Descriptive statistics and Pearson correlation were initially used to examine the relationships between childhood trauma, personality traits, resilience, and depression. To examine the mediating role of resilience in the relationship between childhood trauma and depressive symptoms, a two-step multiple regression analysis approach was employed [39]. First, the effect of childhood trauma on resilience (path a) was assessed. Next, the impact of childhood trauma and resilience on depressive symptoms (path b) was evaluated. The significance of the indirect effect was tested using the Bootstrap method with 5000 resamples; mediation was deemed significant if zero was not within the confidence intervals. All analyses were conducted using SPSS version 22.0, with the significance level set at *p* < 0.05.

In the third phase, LPA was used to identify distinct personality profiles. Before conducting LPA, personality trait scores were standardized to Z-scores. The most suitable model was selected based on criteria including:

(1) Lower comparative indices for Akaike Information Criterion (AIC), Bayesian Information Criterion (BIC), and Sample Size-Adjusted Bayesian Information Criterion (SSABIC).

(2) Statistical significance in Lo-Mendell-Rubin Likelihood Ratio (LMR-LRT) and Bootstrap Likelihood Ratio (BLRT) tests.

(3) Higher entropy values [40].

Ultimately, comparative model analyses and multi-group investigations were conducted to examine the influence of personality types on the interconnections among these variables.

## Results

### Common Method Deviation Control and Inspection

This study employed a self-report scale for data collection, controlling for common method bias by using anonymous assessments, setting a reasonable order for the questions, and appropriately adjusting the questionnaire length. Before data processing, a common method bias test was conducted. Harman’s one-way test extracted 26 factors with eigenvalues >1, cumulatively explaining 60.89% of the variance. The variance explained by the first common factor was 16.54%, which was below the critical threshold of 40% [41], suggesting the absence of significant common method bias in this research.

### Correlation Analysis among Variables

Table 1 presents the correlation coefficients, mean values (M), and standard deviations (SD) for all variables involved in this study. The findings indicate a positive correlation between childhood trauma and depression and a negative correlation between resilience and depression.

**Table 1.** **Correlation coefficients among variables**.

Variable	M	SD	1	2	3	4	5	6	7	8	9	10	11	12	13
1	6.57	2.45	1												
2	5.56	1.73	0.53^**^	1											
3	5.47	1.52	0.37^**^	0.33^**^	1										
4	9.23	3.98	0.41^**^	0.30^**^	0.14^**^	1									
5	8.20	2.78	0.34^**^	0.29^**^	0.25^**^	0.45^**^	1								
6	35.03	8.78	0.74^**^	0.63^**^	0.48^**^	0.79^**^	0.72^**^	1							
7	35.40	6.11	0.03	0.03	0.05	–0.05	–0.09^**^	–0.03	1						
8	32.27	5.33	–0.18^**^	–0.08^*^	–0.05	–0.10^**^	–0.11^**^	–0.15^**^	0.26^**^	1					
9	27.62	5.22	–0.07^*^	–0.04	0.004	–0.16^**^	–0.13^**^	–0.14^**^	0.32^**^	0.23^**^	1				
10	37.40	4.75	–0.15^**^	–0.09^**^	–0.04	–0.20^**^	–0.19^**^	–0.21^**^	0.15^**^	0.27^**^	0.18^**^	1			
11	27.57	5.24	0.22^**^	0.15^**^	0.12^**^	0.17^**^	0.18^**^	0.24^**^	–0.23^**^	–0.43^**^	–0.39^**^	–0.50^**^	1		
12	62.92	14.79	–0.18^**^	–0.09^**^	–0.06^*^	–0.29^**^	–0.25^**^	–0.29^**^	0.43^**^	0.48^**^	0.43^**^	0.30^**^	–0.46^**^	1	
13	18.86	7.60	0.32^**^	0.18^**^	0.19^**^	0.15^**^	0.23^**^	0.30^**^	–0.06^*^	–0.27^**^	–0.16^**^	–0.26^**^	0.46^**^	–0.28^**^	1

**Note**: 1, emotional abuse; 2, physical abuse; 3, sexual abuse; 4, emotional neglect; 5, physical neglect; 6, total childhood trauma score; 7, openness; 8, conscientiousness; 9, extraversion; 10, agreeableness; 11, neuroticism; 12, resilience; 13, depression. ^*^p < 0.05, ^**^p < 0.01. M, mean; SD, standard deviations.

### Mediating Effects of Resilience

**Fig. 1.**
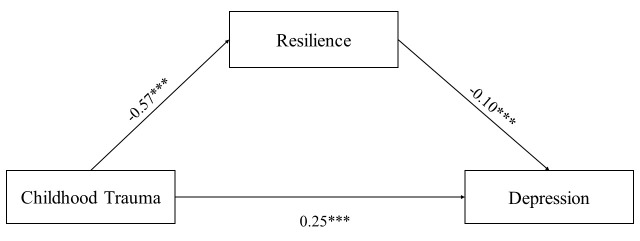
**The mediating role of resilience between childhood trauma and the onset of depression**.^*⁣**^p < 0.001.

The SPSS macro Model 4 was implemented to evaluate the mediation framework, highlighting the intermediary role of resilience between childhood trauma and depressive symptoms. Gender and age were used as covariates in the data analysis. As shown in Table 2, the direct effect of childhood trauma on depression in early adulthood was significant when resilience was included in the regression equation (B = 0.25, *p* < 0.001). Exposure to childhood trauma was inversely related to resilience (B = –0.57, *p* < 0.001), and resilience was a negative predictor of early adult depression (B = –0.10, *p* < 0.001) (Fig. 1).

**Table 2.** **Standardized direct and indirect effects in the model**.

Outcome		Effect	SE	95% CI	*t*-value	*p*-value
Depression	Indirect effect	0.06^*⁣**^	0.01	(0.04, 0.09)	6.00	<0.001
	Direct effect	0.25^*⁣**^	0.03	(0.19, 0.31)	7.91	<0.001
	Total effect	0.30^*⁣**^	0.03	(0.25, 0.36)	10.28	<0.001

**Note**: Path coefficients were derived from a series of regression analyses, and the significance of these coefficients was tested using a two-tailed *t*-test. The *t*-value was calculated based on the coefficient, standard error, and the sample size. Bootstrap sample size = 5000. ^*⁣**^p < 0.001. CI, confidence interval; SE, standard error.

In terms of indirect effects, we identified an indirect pathway of the effect of childhood trauma on depression, specifically: childhood trauma → resilience → depression. The indirect effect was 0.06 (95% confidence interval (CI) = [0.04, 0.09]), the direct effect was 0.25 (95% CI = [0.19, 0.31]), and the total effect was 0.30 (95% CI = [0.25, 0.36]). Indirect effects explained 20% of the total effect. These results suggest that childhood trauma can directly or indirectly exacerbate depressed mood in early adulthood. Consequently, these findings support Hypothesis 2.

### Personality Types

We employed the latent profile analysis (LPA) to categorize individuals into personality types based on the Big Five personality traits. As shown in Table 3, entropy values >0.8 indicate that at least 90% of the classifications were accurate, while values <0.6 indicate that more than 20% of individuals had categorization errors, with higher values representing more accurate categorization [42]. The entropy value of the two-class model was 0.6, and the AIC, BIC, and Adjusted Bayesian Information Criterion (ABIC) values decreased with an increasing number of classifications. The *p*-values of the Class 3 model LMR and BLRT were significant (*p* < 0.05), indicating that the class 3 model is superior to the two-class model. The four-class model exhibited reduced AIC, BIC, and sample-size-adjusted BIC values compared to the three-class model. However, a study has shown that the minimum number of participants within each subgroup should not fall below 5% of the total sample size or be fewer than 30 individuals [43]. One class in the four-class model (n = 29) did not meet this requirement. The entropy value of the five-class model was smaller than that of the three-class and four-class models. Thus, considering model simplicity, we chose the three-class model (Table 3).

**Table 3.** **Evaluation of model fit for five latent profile analysis models (n = 1059)**.

	AIC	BIC	ABIC	*p*LMR	*p*BLRT	Entropy	Group size for each class				
							1	2	3	4	5
1-Class	15,041.557	15,091.208	15,059.446	-	-	-	1059				
2-Class	14,511.275	14,590.716	14,539.898	<0.0001	<0.0001	0.600	563	496			
3-Class	14,334.215	14,443.446	14,373.571	0.0007	<0.0001	0.714	705	137	217		
4-Class	14,279.386	14,418.409	14,329.476	0.0411	<0.0001	0.721	29	331	116	583	
5-Class	14,229.034	14,397.847	14,289.857	0.0138	<0.0001	0.677	44	100	265	101	549

**Note**: AIC, Akaike Information Criterion; BIC, Bayesian Information Criterio; ABIC, Adjusted Bayesian Information Criterion; *p*LMR, The *p*-value of Lo-Mendell-Rubin; *p*BLRT, The *p*-value of Bootstrap Likelihood Ratio Tests.

**Fig. 2.**
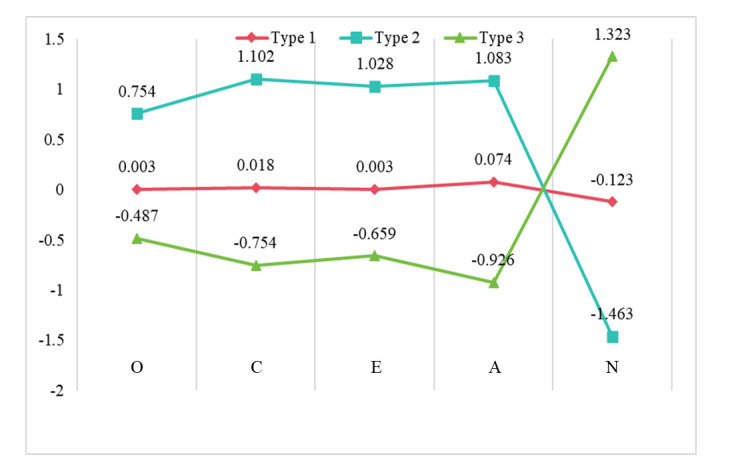
**The underlying pattern of personality types among college students**. This chart shows the scores of three personality types (Type 1, Type 2, Type 3) on the five personality dimensions of openness (O), conscientiousness (C), extroversion (E), agreeableness (A), and neuroticism (N).

In this study, Chinese university students were classified into three personality types based on the following criteria: z-scores equal to or greater than 0.5 represent high scores, z-scores between –0.5 and 0.5 represent moderate scores, and z-scores equal to or less than –0.5 represent low scores (Table 4). Consequently, Type 1 (n = 705, 66.57%) was characterized by moderate openness, conscientiousness, extraversion, agreeableness, and neuroticism; Type 2 (n = 137, 12.94%) was characterized by high openness, conscientiousness, extraversion, agreeableness, and low neuroticism; and Type 3 (n = 217, 20.49%) was characterized by moderate scores for openness, low scores for conscientiousness, extroversion, and agreeableness, and high neuroticism (Fig. 2). According to Robins *et al*. [24], Type 2 personality is resilient, characterized by higher-than-average values of openness, conscientiousness, extraversion, and agreeableness, and the lowest neuroticism. By contrast, Type 1 and Type 3 personalities do not precisely replicate the over- and under-controlled types from the RUO personality type model.

**Table 4.** **Standard scores of the three personality types on the five personality traits**.

Trait	O	C	E	A	N
Type 1	0.003	0.018	0.003	0.074	–0.123
Type 2	0.754	1.102	1.028	1.083	–1.463
Type 3	–0.487	–0.754	–0.659	–0.926	1.323

**Note**: O, openness; C, conscientiousness; E, extraversion; A, agreeableness; N, neuroticism. Type 1 (n = 705, 66.57%): Medium openness, conscientiousness, extroversion, agreeableness and neuroticism. Type 2 (n = 137, 12.94%): High openness, conscientiousness, extroversion, agreeableness; Low neuroticism. Type 3 (n = 217, 20.49%): Medium openness; Low conscientiousness, extroversion, agreeableness; High neuroticism.

A one-way ANOVA was conducted with personality type as the independent variable and childhood trauma, resilience, and depression as the dependent variables (Table 5). The results indicate significant differences among the personality types at these three dependent variables (*p* < 0.001).

**Table 5.** **Average values and variability measures for each personality category**.

	Childhood trauma M ± SD	Resilience M ± SD	Depression M ± SD
Type 1 (n = 705)	1.38 ± 0.31a	2.56 ± 0.50b	0.89 ± 0.33c
Type 2 (n = 137)	1.26 ± 0.29b	3.07 ± 0.53c	0.74 ± 0.28a
Type 3 (n = 217)	1.55 ± 0.46c	2.03 ± 0.53a	1.24 ± 0.44b
*F*	33.48^*⁣**^	179.37^*⁣**^	108.44^*⁣**^

**Note**: Mean (M) and standard deviation (SD) of childhood trauma, resilience, and depression. The different letters in each column (a, b, c) represent statistically significant differences between the pairwise corresponding variables in the three personality types. Specifically, if the mean values of the variables between the two personality types are marked with different letters, it means that there is a statistically significant difference between them. (***p < 0.001).

### The Moderating Role of Personality Type

Through model comparisons and multi-group analyses, we identified a moderating effect of personality type. We added the personality type variable to Model 1 and examined its effect.

#### The Effect of Personality Type on the Relationship Linking Childhood Trauma, through Resilience, to Depression

**Fig. 3.**
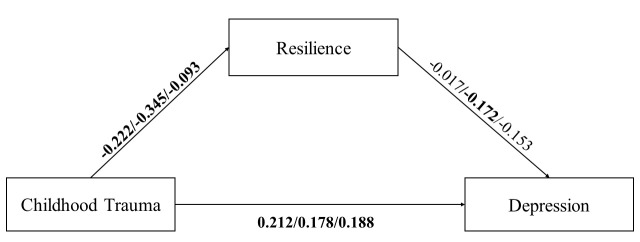
**Mediation effect analysis with depression as the outcome variable for Type 1, Type 2, and Type 3 personalities**. Path coefficients in bold indicate statistical significance at *p* < 0.05.

Fig. 3 illustrates the outcomes of the mediation effect analysis for each personality type (Table 6). For Type 1 personality, childhood negatively influenced resilience (*B *= –0.222, *p* < 0.001, 95% CI = [–0.29, –0.15]) and positively predicted depression (B = 0.212, *p* < 0.001, 95% CI = [0.14, 0.29]), whereas the negative correlation between resilience and depression was not significant (B = –0.0173, *p* = 0.654, 95% CI = [–0.09, 0.06]). For Type 2 personality, childhood trauma had a detrimental impact on resilience (B = –0.345, *p* < 0.001, 95% CI = [–0.52, –0.17]) and positively predicted depression (B = 0.178, *p* = 0.0239, 95% CI = [0.02, 0.33]), while resilience was significantly negatively correlated with depression (B = –0.172, *p* = 0.0175, 95% CI = [–0.31, –0.03]). For Type 3 personality, childhood trauma negatively predicted resilience (B = –0.093, *p* =0.0419, 95% CI = [–0.18, –0.002]) and positively predicted depression (B = 0.188, *p* = 0.0026, 95% CI = [0.07, 0.30]), while the negative correlation between resilience and depression was not significant (B = –0.153, *p* = 0.0749, 95% CI = [–0.32, 0.02]).

**Table 6.** **Regression analysis of mediating effects of childhood trauma and resilience on depression**.

	Childhood Trauma → Resilience					Resilience → Depression					Childhood trauma → Depression				
	B	SE	95 % CI	*t*	*p*	B	SE	95% CI	*t*	*p*	B	SE	95 % CI	*t*	*p*
Type 1	–0.222^*⁣**^	0.037	(–0.29, –0.15)	–6.085	<0.001	–0.0173	0.039	(–0.09, 0.06)	–0.449	0.654	0.212^*⁣**^	0.038	(0.14, 0.29)	5.556	<0.001
Type 2	–0.345^*⁣**^	0.090	(–0.52, –0.17)	–3.841	<0.001	–0.172^*^	0.072	(–0.31, –0.03)	–2.407	0.0175	0.178^*^	0.078	(0.02, 0.33)	2.285	0.0239
Type 3	–0.093^*^	0.046	(–0.18, –0.002)	–2.005	0.0419	–0.153	0.086	(–0.32, 0.02)	–1.782	0.0749	0.188^**^	0.059	(0.07, 0.30)	3.196	0.0026

**Note**: All variables in the model were standardized and included in the regression equation. ^*^p <*0*.05, ^**^p < 0.01, ^*⁣**^p < 0.001.

Under the influence of the three personality types, the direct and indirect effects of all mediating paths differed from those observed in Model 1. The indirect effects of the mediating pathways for any of the three personality types were not significant (Table 7).

**Table 7.** **Direct and indirect effects on different personality models**.

Outcome		Effect	SE	95% CI	*t*-value	*p*-value
Type 1	Indirect effect	0.0038	0.010	(–0.156, 0.023)	0.38	0.704
	Direct effect	0.2115^*⁣**^	0.038	(0.137, 0.286)	5.56	<0.001
	Total effect	0.2154^*⁣**^	0.037	(0.143, 0.288)	5.81	<0.001
Type 2	Indirect effect	0.0594	0.038	(–0.002, 0.146)	1.563	0.118
	Direct effect	0.1778^*^	0.078	(0.024, 0.332)	2.285	0.024
	Total effect	0.2372^**^	0.0751	(0.089, 0.386)	3.158	0.002
Type 3	Indirect effect	0.0143	0.0124	(–0.0023, 0.045)	1.153	0.249
	Direct effect	0.1881^**^	0.059	(0.072, 0.304)	3.196	0.002
	Total effect	0.2023^**^	0.059	(0.087, 0.318)	3.45	0.001

**Note**: The path coefficients were derived from a series of regression analyses, and the significance of these coefficients was tested using a two-tailed *t*-test. The *t* value was calculated based on the coefficient, standard error, and the sample size. Bootstrap sample size = 5000. ^*^p < 0.05, ^**^p < 0.01, ^*⁣**^p < 0.001.

## Discussion

### Reproducibility of RUO Personality Types

This study applied a typological approach to personality research. Based on our hypotheses, LPA clustering identified three optimal personality clusters within this sample. The resilient personality type could be replicated in the three-cluster solution, whereas Types 1 and 3 were not exact replicas of the under- or over-controlled personality types. These findings are consistent with the prior study, indicating that the RUO class is only partially replicable in a predominantly Chinese sample from Hong Kong [44]. Our findings suggest that cultural context must be considered when studying the links between mental health and personality.

### The Mediating Role of Resilience

Previous studies corroborate the outcomes of this research, indicating that resilience is a partial mediator in the connection between childhood trauma and depressive symptoms. Resilience has also been identified to moderate this relationship [17]. Findings from a meta-analytic review demonstrate that while resilience plays a significant mediating role in the link between trauma and depression, there is no notable distinction in depressive symptoms among individuals with a trauma history based on their levels of resilience [11]. Examining this through the lens of personality traits, existing literature establishes that neuroticism is a mediator between childhood trauma and depressive symptoms among college students, while resilience moderates the relationship between childhood trauma and neuroticism [45].

### Limitations and Prospects

This study has several limitations that suggest directions for future research. First, it utilized a self-reported questionnaire, which may have introduced response bias despite providing standard quantitative metrics. The CES-D scale measures depressive symptoms but does not diagnose clinical depression. The high incidence of depression reported in this study reflects transient symptoms and should not be conflated with clinical diagnoses. Future investigations could benefit from experimental research designs or diverse sources and methodologies for data collection. Using the Big Five personality dimensions, this study classified each personality type into its corresponding RUO category. However, gaps and inconsistencies in the literature led to the inability of the sample to distinguish between under-controlled and over-controlled personality types. Future research should address the absence of standardized procedures for recognizing RUO types. Additionally, this study employed a cross-sectional design. Future research should use a longitudinal design to examine the pathways of influence linking variables more explicitly and to further explore the effects of childhood trauma on depression in early adulthood.

## Conclusions

This research explores the relationship between childhood trauma and depressive symptoms, investigating the mediating role of resilience and the moderating influence of personality types. The study reports a positive correlation between childhood trauma and depressive symptoms in early adulthood, with resilience significantly mediating this relationship, especially for Type 2 personalities in the RUO model. These findings highlight the significance of integrating personality and resilience in mental health strategies and clinical practice, advocating for personalized approaches to foster resilience and address the impact of childhood trauma on depression. The research opens avenues for further exploration of resilience and personality in preventive and intervention strategies in mental health care.

## Availability of Data and Materials

The data that support the findings of this study are openly available in [“Psychological Science Data Bank”] at [https://doi.org/10.57760/sciencedb.psych.00266].
